# Supplementary material for: Biotransformation of Chlorpyrifos Shewanella oneidensis MR-1 in the Presence of Goethite: Experimental Optimization and Degradation Products
Source: Toxics. 2024 May 31;12(6):402. doi: 10.3390/toxics12060402 (PMC11209498; doi:10.3390/toxics12060402)
Supplement: Supplementary file 1 [file toxics-12-00402-s001.zip › toxics-3023503-supplementary.pdf]

# Supplementary Material

Chlorpyrifos and its metabolites were determined by Agilent 1290 Infinity UPLC liquid chromatography system and Agilent 6550 iFunnel Q-TOF mass spectrometry. The total running time of liquid chromatography was 15 min, the injection volume was 5 μL, and the flow rate was 0.3ml/min. A Waters BEH C18 2.1×50mm (1.7 μm) column was used for separation. The analyte was ionized by dynamic multiple reaction monitoring (MRM) using an electrospray ionization source. The MS parameters were set as follows: sheath temperature was 350°C and sheath gas flow rate was 12 L/min; The ESI+ mode voltage is 4000V, and the ESI- mode voltage is 3200V. Ms scanning range: m/z = 50-600. The gradient leaching procedure is shown in T**able S1**.

**Table S1. Gradient drenching scheme**

| Time (min) | **Phase A** (%) 0.1% Formic Acid in Ultrapure water | **Phase B** (%) Methanol |
|---|---|---|
| 0 | 95 | 5 |
| 1 | 95 | 5 |
| 9 | 0 | 100 |
| 13 | 0 | 100 |
| 14 | 95 | 5 |
| 15 | 95 | 5 |

**Table S2.** The location and assignments of FT-IR peaks.

| Number | Location ($cm^{-1}$) | Assignments | References |
|---|---|---|---|
| 1 | 3000~3500 | -OH deformation band | (M. Yang et al., 2021; Zhu et al., 2019) |
| 2 | 1645 | C=O stretching vibration | Elzinga et al., 2012 |
| 3 | 1360-1450 | $\upsilon_{S(-COO)}$ | Elzinga et al., 2012 |
| 4 | 1051 | C-OH, C-O-C, and C-C vibrations of polysaccharides | Elzinga et al., 2012 |
| 5 | 891 | σ-OH | Oliveira et al., 2008 |
| 6 | 796 | γ-OH | Oliveira et al., 2008 |
| 7 | 637 | Fe-O | Oliveira et al., 2008 |

**Table S3.** XPS C1s fitting parameters of goethite before and after reactions.

| Degradation stage | | C-C/C=C | C-OH | C=O | O-C=O |
|---|---|---|---|---|---|
| 2 Day | B. E. (eV) | 284.64 | 285.63 | 287.02 | 288.80 |
| | Per. (%) | 33.49 | 21.28 | 5.71 | 1.81 |
| 10 Day | B. E. (eV) | 283.82 | 285.40 | 287.00 | / |
| | Per. (%) | 8.55 | 7.47 | 2.89 | / |

**Table S4.** XPS O1s fitting parameters of Goethite before and after reactions.

| Degradation stage | | Fe-O | Fe-OH | $H_2O$ |
|---|---|---|---|---|
| 2 Day | B. E. (eV) | 529.86 | 531.73 | 533.48 |
| | Per. (%) | 2.77 | 15.17 | 5.03 |
| 10 Day | B. E. (eV) | 530.36 | 529.06 | 531.73 |
| | Per. (%) | 26.29 | 15.82 | 10.89 |

**Table S5.** XPS Fe (III) fitting parameters of goethite before and after reactions.

| Degradation stage | | Fe (III) | | | | | |
|---|---|---|---|---|---|---|---|
| | | Fe $2p_{3/2}$ | Fe $2p_{1/2}$ | Fe $2p_{3/2}$ | Fe $2p_{1/2}$ | Satellite 1 | Satellite 2 |
| 2 Day | B. E. (eV) | 710.88 | 724.18 | 712.78 | 725.62 | 719.88 | / |
| | Per. (%) | 0.70 | 0.41 | 0.43 | 0.43 | 0.06 | / |
| 10 Day | B. E. (eV) | 710.85 | 724.33 | 713.41 | 726.39 | 718.68 | 732.37 |
| | Per. (%) | 3.66 | 4.90 | 3.26 | 3.24 | 1.61 | 0.21 |

**Table S6.** XPS Fe (II) fitting parameters of goethite before and after reactions.

| Degradation stage | | Fe (II) | | | | | |
|---|---|---|---|---|---|---|---|
| | | Fe $2p_{3/2}$ | Fe $2p_{1/2}$ | Fe $2p_{3/2}$ | Fe $2p_{1/2}$ | Satellite 1 | Satellite 2 |
| 2 Day | B. E. (eV) | / | / | / | / | 719.88 | / |
| | Per. (%) | / | / | / | / | 0.06 | / |
| 10 Day | B. E. (eV) | 709.54 | 722.75 | / | / | 718.68 | 732.37 |
| | Per. (%) | 3.94 | 3.03 | / | / | 1.61 | 0.21 |
